# Challenges with cataract surgery in pars planitis patients

**DOI:** 10.1007/s00417-017-3698-6

**Published:** 2017-06-07

**Authors:** Andrzej Grzybowski, Piotr Kanclerz, Uwe Pleyer

**Affiliations:** 1 Department of Ophthalmology, Poznan City Hospital, ul. Szwajcarska 3, 61-285 Poznan, Poland; 2 Department of Ophthalmology, University of Warmia and Mazury, Olsztyn, Poland; 3 Department of Ophthalmology, Medical University of Gdańsk, Gdańsk, Poland; 4 Department of Ophthalmology, Charité, University Medicine Berlin, Campus Virchow-Klinikum, Berlin, Germany

Children and teens with intraocular inflammation often present with cataract. It has been predicted that the risk for cataract development in children with anterior uveitis and juvenile idiopathic arthritis (JIA) is 0.16 events per patient-year of follow-up [1]. This is likely also the case for pars planitis. Pars planitis, which presents mainly in younger ages, is a subset of intermediate uveitis characterized by the presence of snowbanks at the pars plana and inflammatory cells in the vitreous. We often realize that these patients have worse vitritis, more severe macular edema and a guarded prognosis when compared to our other patients with intermediate uveitis. Indeed, cataract formation is the most frequent cause of severe vision loss in pediatric patients suffering from pars planitis, followed by vitreous opacification [2, 3]. The close proximity of inflammatory changes at the pars plana to the lens with a constant accumulation of immune mediators, as well as long term corticosteroid treatment, predispose these eyes for cataract development [4, 5].

Whereas adult uveitic cataracts are managed with phacoemulsification with primary intraocular lens (IOL) implantation, no standard approach exists for children. At a time when cataract surgery is a daily routine in young patients as well as adults, this may not appear as a major task. In the context of uveitis, however, it remains a challenge. It requires not only a meticulous procedure, but also critical timing of intervention and a deeper understanding of the underlying inflammatory mechanisms. The question of whether an IOL can be tolerated in uveitic eyes has been disputed for decades. It is now mainly agreed that an IOL can be safely implanted in Fuchs uveitic and idiopathic nongranulomatous eyes, whereas moderate success might be expected, e.g. in Behcets disease and HLA-B27 associated uveitis, still the most critical patients remain young children with juvenile idiopathic arthritis [6–9]. It is uncertain, however, which type of IOL provides the best visual outcome in uveitic eyes [10]. This issue was meticulously investigated by Leung et al. in the Cochrane Database for Systematic Reviews. Current evidence supports a superior effect of hydrophobic acrylic lenses over silicone lenses, specifically for posterior synechiae outcomes [11].

Since both cataract and vitreous haze affect vision in pars planitis, combined procedures can be considered. The question of simultaneous cataract extraction and vitrectomy has so far rarely been addressed. Is this additional effort worthwhile?

Albavera-Giles et al. have addressed the question in this issue of the journal [12]. More than two-thirds of all their cataract interventions were performed as combined procedures including vitrectomies. Therefore, this work differs in several points from previous observations.

Firstly, the combined procedure is a relatively “aggressive” approach, given the relatively young age of the cohort under investigation. It has to be stressed that these eyes often present with a small pupil, anterior and/or posterior synechiae, cyclitic membranes and even zonulolysis. Consequently, this may lead to an undersized or incomplete capsulorhexis, iris prolapse, increased risk of posterior capsular tear, retained nuclear fragments and increased risk of intraoperative zonular dehiscence [13]. In addition, it has to be taken in mind that in these young patients a tight and adherent vitreous bears higher risks for retina complications [14].

Second, the results are at first sight somewhat sobering. Although a visual gain of two lines was achieved in 60% of all eyes, only four achieved a visual acuity of 20/40 or better. More favorable results have already been reported [6, 7, 9]. However, a closer look indicates that amblyopia and macular edema were among the most frequent causes to explain this outcome. It is noteworthy that, despite the young age of the patients and the challenging initial situations, no major complications occurred.

Here the third and presumably co-determining factor with paramount importance comes into consideration. The authors have consistently focused on inflammatory sequelae before the intervention, with a close look to controlling inflammation. It has been reiterated many times that the most important step in cataract surgery in uveitic eyes is adequate pre-operative control of inflammation. The agent of choice in this study was methotrexate (MTX) and used in almost all patients. Even when it may not be the most potent agent to control posterior segment inflammation, the clinical experience with MTX in children is favorable. Its use in childhood uveitis is based on a number of case series and meta-analyses, but no randomized controlled trial has been performed [15–17]. Since it is applied only once a week, it enhances patient’s adherence. Its long-term safety profile is well recognized and particular important in young patients. Other immunomodulatory treatments have been used with variable success, including antimetabolites (azathioprine), calcineurin inhibitors (cyclosporine A, tacrolimus), and biologics, e.g. tumor necrosis factor-alpha antagonists (infliximab, adalimumab) and interleukin-6 antagonists (tocilizumab) [16, 18]. It remains speculative whether a more specific treatment, for example with a biologic agent, would have shown a more favorable outcome in this study.

In summary, adequate preoperative control of inflammation, a meticulous surgical technique, a foldable hydrophobic acrylic intraocular lens implanted in the capsular bag and good control of postoperative inflammation are crucial for successful cataract surgery in pars planitis patients.
